# Characterization of a new simian immunodeficiency virus strain in a naturally infected *Pan troglodytes troglodytes *chimpanzee with AIDS related symptoms

**DOI:** 10.1186/1742-4690-8-4

**Published:** 2011-01-13

**Authors:** Lucie Etienne, Eric Nerrienet, Matthew LeBreton, Godwin Tafon Bibila, Yacouba Foupouapouognigni, Dominique Rousset, Ahmadou Nana, Cyrille F Djoko, Ubald Tamoufe, Avelin F Aghokeng, Eitel Mpoudi-Ngole, Eric Delaporte, Martine Peeters, Nathan D Wolfe, Ahidjo Ayouba

**Affiliations:** 1 UMR145, Institut de Recherche pour le Développement (IRD) and Université Montpellier 1, Montpellier, France; 2 HIV laboratory, Centre Pasteur du Cameroun, Yaounde, Cameroon; 3 HIV/Hepatitis Laboratory, Pasteur Institute, Phnom Penh, Kingdom of Cambodia; 4 Global Viral Forecasting (GVF), San Francisco, USA; 5 Apes Action Africa, Yaoundé, Cameroon; 6 Virology Laboratory CRESAR/IMPM/IRD, Yaoundé, Cameroon; 7 Stanford University, Program in Human Biology, Stanford, California, USA

## Abstract

**Background:**

Data on the evolution of natural SIV infection in chimpanzees (SIVcpz) and on the impact of SIV on local ape populations are only available for Eastern African chimpanzee subspecies (*Pan troglodytes schweinfurthii*), and no data exist for Central chimpanzees (*Pan troglodytes troglodytes*), the natural reservoir of the ancestors of HIV-1 in humans. Here, we report a case of naturally-acquired SIVcpz infection in a *P.t.troglodytes *chimpanzee with clinical and biological data and analysis of viral evolution over the course of infection.

**Results:**

A male chimpanzee (Cam155), 1.5 years, was seized in southern Cameroon in November 2003 and screened SIV positive during quarantine. Clinical follow-up and biological analyses have been performed for 7 years and showed a significant decline of CD4 counts (1,380 cells/mm^3 ^in 2004 vs 287 in 2009), a severe thrombocytopenia (130,000 cells/mm^3 ^in 2004 vs 5,000 cells/mm^3 ^in 2009), a weight loss of 21.8% from August 2009 to January 2010 (16 to 12.5 kg) and frequent periods of infections with diverse pathogens.

DNA from PBMC, leftover from clinical follow-up samples collected in 2004 and 2009, was used to amplify overlapping fragments and sequence two full-length SIVcpz*Ptt*-Cam155 genomes. SIVcpz*Ptt*-Cam155 was phylogenetically related to other SIVcpz*Ptt *from Cameroon (SIVcpz*Ptt*-Cam13) and Gabon (SIVcpz*Ptt*-Gab1). Ten molecular clones 5 years apart, spanning the V1V4 gp120 *env *region (1,100 bp), were obtained. Analyses of the *env *region showed positive selection (dN-dS >0), intra-host length variation and extensive amino acid diversity between clones, greater in 2009. Over 5 years, N-glycosylation site frequency significantly increased (p < 0.0001).

**Conclusions:**

Here, we describe for the first time the clinical history and viral evolution of a naturally SIV infected *P.t.troglodytes *chimpanzee. The findings show an increasing viral diversity over time and suggest clinical progression to an AIDS-like disease, showing that SIVcpz can be pathogenic in its host, as previously described in *P.t.schweinfurthii*. Although studying the impact of SIV infection in wild apes is difficult, efforts should be made to better characterize the pathogenicity of the ancestors of HIV-1 in their natural host and to find out whether SIV infection also plays a role in ape population decline.

## Background

While non-invasive studies have provided a clear picture on the prevalence and genetic diversity of simian immunodeficiency virus (SIV) infection in wild apes in Central Africa and allowed the tracing of the origins of human immunodeficiency virus type 1 (HIV-1) infection in humans, there is almost no information on clinical, immunological, and intra-host viral evolution for natural SIV infections in chimpanzees and gorillas. Studying SIV infection over time in apes is not facilitated by their isolated habitat and endangered status. While non-invasive studies could potentially allow evaluation of viral evolution over time, they cannot yet provide information on the clinical history of the animal. Follow-up studies are thus performed on captive animals, but only a handful of captive chimpanzees with natural SIVcpz infections have been identified (Additional file 1: Table S1), and no captive SIV infected gorilla has been described. To date, six strains of SIVcpz*Ptt *were characterised in captive *Pan troglodytes troglodytes *chimpanzees, but no virological or clinical follow-up data were available because the animals died upon arrival, were of a young age, or were tested retrospectively [1-4]. The clinical measurements and disease course for one captive SIV positive *Pan troglodytes ellioti *(Cam4) were described, but this animal most likely acquired his infection in captivity from his naturally infected *P. t. troglodytes *cage mate (Cam3); no natural SIVcpz infection has been identified in wild animals from *P. t. ellioti *[1,5]. Finally, a confiscated *Pan troglodytes schweinfurthii *chimpanzee [6], rescued following illegal export from Africa to Belgium, is currently the only naturally SIVcpz infected chimpanzee known to be alive. This animal is infected with SIVcpz*Pts*-Ant, and had been regularly monitored over a 7-year period from the ages of 4 to 11 years old. This observation period has provided unique data on virological and immunological characteristics of a natural SIVcpz*Pts *infection [7-9]; the SIVcpz*Pts*-Ant strain showed an important genetic variability in V1 and V2 *env *regions and the animal presented no signs of immunodepression with a strong humoral antibody response, fluctuating plasma viremia, and a strong but transient neutralizing antibody response [8]. Nevertheless, the animal's platelet count eventually dropped to extremely low values at age 7, leading to a profound and permanent thrombocytopenia [10], a characteristic that has been observed and associated with progressive HIV and SIV infections in humans and pigtail macaques, respectively [11,12].

Recently, the paradigm that SIVs are non-pathogenic in their natural hosts has been challenged for chimpanzees [13]. It was shown on habituated communities of wild *P. t. schweinfurthii *chimpanzees in Gombe, Tanzania, that SIVcpz*Pts *infection is associated with a 10 to 16 fold increase in age-corrected risk for death, reduced fertility in SIV positive females in terms of birth rate and survival of off-spring, and an AIDS (acquired immune deficiency syndrome)-like syndrome which correlated with low CD4 counts, revealed by post-mortem immunohistochemistry. Thus, these observations suggest that SIVcpz has a similar effect on chimpanzees as HIV-1 has on humans.

Chimpanzees are also the only animals that can be experimentally infected with HIV-1; they are readily susceptible to HIV-1 but, in contrast to humans, the infection generally does not progress to AIDS. Over 100 chimpanzees have been infected with HIV-1, but only a few cases of immune deficiency were reported, all occurring in the Yerkes cohort [14-17]. It has to be noted that chimpanzees used for the experiments were almost all from the *P. t. verus *subspecies from West Africa, in which no natural SIV infection has been documented to date.

Today, data on the evolution of natural SIVcpz infection are only available from a single chimpanzee, and no data exist for representatives of the *P. t. troglodytes *subspecies in which the reservoir of the ancestors of HIV-1 in humans has been identified. Here, we report a new case of a natural SIVcpz*Ptt *infection in a *P. t. troglodytes *chimpanzee (Cam155/Ch-Go) from Cameroon; we characterized the full-length genome and analyzed the viral diversity and evolution of the SIVcpz*Ptt*-Cam155 strain at a five-year interval. Importantly, the clinical and biological data recorded on this chimpanzee following his arrival at the sanctuary suggest progression to AIDS.

## Results

### Clinical history and observations of the SIVcpz*Ptt* infected animal, Cam155

Cam155 (Ch-Go) is a male chimpanzee that arrived in the sanctuary in Cameroon, in November 2003 at approximately 1.5 years old. The animal was confiscated by the Ministry of Environment and Forestry near the Dja Faunal Reserve in south-central Cameroon, located within the natural range and habitat of *P. t. troglodytes*. Upon arrival, the animal was emaciated, dehydrated, and had wounds to the groin (Table 1). During his quarantine in December 2003, Ch-Go was screened for SIV infection and had a positive reaction in HIV screening and confirmatory tests. Serological tests for other viral infections, such as hepatitis A, B and C viruses, and simian T-lymphotropic virus, were all negative, and there was no evidence of tuberculosis infection.

**Table 1:** Clinical history of the SIVcpz*Ptt*-Cam155 infected chimpanzee (Ch-Go) since his arrival at the sanctuary in November 2003.

Year	Months	Symptoms	Viral load **(Log copies/ml)**^**a**^	CD4/CD8 counts **(cells/mm**^**3**^**)**^**b**^	Platelets **(cells/mm**^**3**^**)**	Weight (kg)	Age (years)
2003	11	Cachexia, dehydratation, wounds to the groin at arrival in sanctuary				3.3	1.5

2004	02	Anorexic		CD4 = 700; CD8 = 570			

	02-04	Respiratory illness					

	03	*Balantidium coli, Entamoeba hartmanni, Trichomonas hominis*	5.09				

	04	*Strongyloides*					

	05			CD4 = 1,380; CD8 = 1,010	130,000		

	06	*Balantidium coli, Ancylostoma*					

2005	10	Cestode infection				8	3.5

2006	02-04	Respiratory illness					

	03	Swelling in eye lid surgically relieved					

	05	Fungal skin infection					

	11	Respiratory illness					

2007	02					14	4.8

2008	01	Oral candidiasis complicated with bacterial infection					

	03	Nose bleeding					

2009	08	An eye infection led to cataract and blindness in one eye		CD4 = 287; CD8 = 1,523		16	7.3

	11	Halitosis, bleeding gums and tooth decay	4.04		5,000		

2010	01	Halitosis, bleeding gums and tooth decay				12.5	7.7

^a ^Viral load was determined with the b-DNA method (Versant HIV-1 RNA 3.0, Siemens, Erlangen, Federal Republic of Germany) in 2004 and with the Abbott RealTime™ HIV-1 assay (Abbott, Chicago, USA) in August 2009.

^b ^CD4 and CD8 counts were determined with Dynabeads in February and May 2004 and with Flow Cytometry in August 2009.

Since his arrival in the sanctuary, the animal has regularly suffered from bite wounds on hands, feet, ears and genitalia from other chimpanzees, as well as from frequent periods of illness. In 2004, various infections with helminths and protozoans were detected (*Balantidium coli, Entamoeba hartmanni*, *Trichomonas hominis, Strongyloides *and *Ancylostoma*), and the animal suffered from an undiagnosed respiratory illness (coughing) (Table 1). Plasma viral load was measured with the commercially available HIV viral load assay in March 2004 (Versant HIV-1 RNA 3.0 (b-DNA), Siemens, Erlangen, Federal Republic of Germany), which revealed a high viral load of 5.09 log_10 _copies/ml. CD4 and CD8 counts were measured in February and May of the same year with Dynabeads technology (Invitrogen, Cergy Pontoise, France) [18,19] and were of 700 and 1,380 CD4 cells/mm^3 ^and 570 and 1,010 CD8 cells/mm^3^, respectively (Table 1). The mean CD4 counts, measured between 2002 and 2004 on 15 SIV negative chimpanzees from the same sanctuary with the same technique, were 1,740 +/- 776, ranging from 540 to 3,460. In 2006, the animal suffered from another unidentified respiratory illness (coughing/catarrh) between February and April, from a swelling in the eyelid in March, from a fungal skin infection in May, and yet another respiratory illness with bilateral nasal discharges, coughing, elevated temperature and mouth breathing in November of the same year. In 2008, oral candidiasis was detected in January and nose bleeding in March. In August 2009, an eye infection (inflammation, weeping and pain) was noted; and, despite treatment attempts, the infection led to cataract and blindness in one eye. Cam155 has experienced growth retardation, and weighed only 16 kg in August 2009, at 7.3 years old, compared to an average weight of 28 kg for four other seven-year old animals in the same sanctuary in Cameroon, and approximately 28-30 kg or more for laboratory raised *P. t. versus *[20,21]. Between August 2009 and January 2010, a significant weight loss (from 16 to 12.5 kg, 21.8%) was observed together with halitosis, bleeding gums and notable tooth decay.

In August 2009, a blood sample was taken to measure CD4/CD8 counts and revealed a significant drop in CD4 counts to 287 cells/mm^3^, CD8 counts were 1,523 cells/mm^3^, a total CD3 average of 1,856 cells/mm^3 ^and CD4/CD8 ratio of 0.19 using a Becton Dickinson FACSCount system (Table 1). By comparison, a healthy seronegative chimpanzee of similar age from the same population had average CD4 counts of 1,256/mm^3 ^and normal chimpanzee values between 800 and 2,000 have also been reported for other captive HIV/SIV negative, asymptomatic experimentally HIV-1 infected chimpanzees and the naturally SIVcpz*Pts*-Ant infected chimpanzee (Ch-No) [22,10].

A commercially available HIV viral load test, Abbott RealTime™ HIV-1 assay (Abbott, Chicago, USA), was used to quantify viral load on a blood sample from November 2009, and plasma viral load was estimated at 10,995 copies/ml (4.04 log_10_). A full blood count in January 2010 revealed slight anaemia (RBC 4,420,000/mm^3 ^compared to 5,040,000 ± 460,000/mm^3^; haemoglobin 10.1 g/dl compared with 13.5 ± 1.2 g/dl and haematocrit 30.33% compared to 41.7 ± 4%); slight leukopenia (8,140/mm^3 ^compared to 13,700 ± 4,600/mm^3^); and severe thrombocytopenia (5,000/mm^3 ^compared with 385,000 ± 77,000/mm^3^) (Table 1). Normal values between brackets correspond to data reported on healthy chimpanzees [23].

Overall, episodes of infections have also been observed in other animals from the sanctuary, but Ch-Go suffered from 8 symptomatic episodes: 4 with parasite infections, 2 with fungal infections, and 2 respiratory illnesses (Table 1). Overall, repeated symptomatic events were rarely seen in the other chimpanzees, especially oral candidiasis together with frequent episodes of eye, respiratory and parasite infections in a single animal was not seen in the other chimpanzees from the same sanctuary. We evaluated the symptoms and clinical history observed in Cam155/Ch-Go using the CDC and WHO classification systems for human HIV infections and found that the disease stage in this animal corresponds at least to pre-AIDS, CDC stage B2 and WHO stage III.

### Full-length genome sequences of the SIVcpz*Ptt*-Cam155 strain

**Figure 1:**
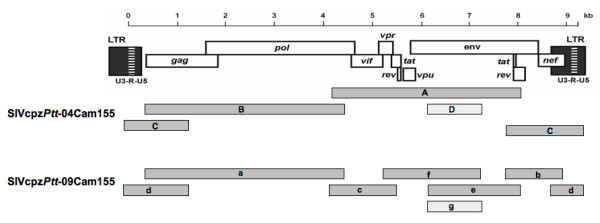
**Amplification of SIVcpz*Ptt*-04Cam155 and SIVcpz*Ptt*-09Cam155 full-length genomes**. The two full-length genomes were amplified as partially overlapping PCR fragments (shaded boxes) and directly sequenced, except for the V1V4 *env *regions (hatched boxes) where clonal sequences were necessary. The primers used to amplify each PCR fragment are given in Additional file 2: Table S2. Fragments are drawn to scale and the nucleotide sequences are numbered from the beginning of the R region in the 5' LTR (see scale bar).

**Figure 2:**
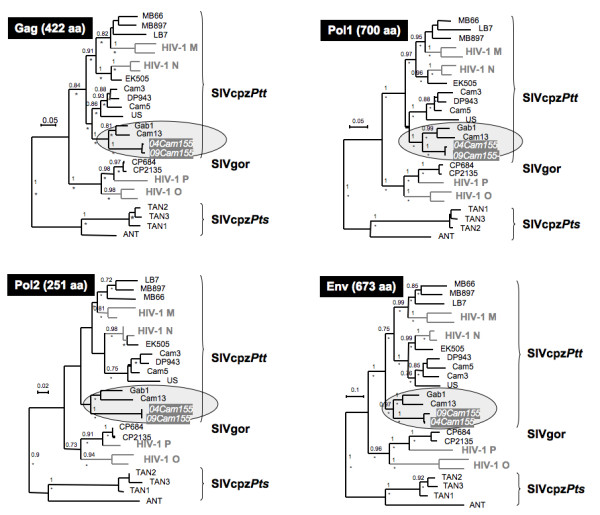
**SIV infecting Cam155/Ch-Go is a SIVcpz*Ptt *closely related to SIVs from Cameroon (SIVcpz*Ptt*-Cam13) and Gabon (SIVcpz*Ptt*-Gab1) across the genome**. SIVcpz*Ptt*-04Cam155 and SIVcpz*Ptt*-09Cam155 Gag, Pol1, Pol2, and Env amino acid (aa) sequences were compared to previously published SIVgor, SIVcpz and HIV-1 references. 422 amino acids were examined for Gag analysis (up left), 700 aa for Pol1 (up right), 251 aa for Pol2 (below left), and 673 aa for Env (below right). Maximum likelihood and Bayesian analysis trees had the same topology. Here presented in black and above the branches, the bootstraps > 0.70, and the grey stars below the branches are posterior probabilities > 0.80. Scale bars represent 0.05 (Gag), 0.05 (Pol1), 0.02 (Pol2) and 0.1 (Env) replacements per site.

DNA extracted from PBMCs obtained from residual blood samples in May 2004 and five years later, in May 2009, was used to generate full-length sequences of the SIVcpz strain infecting Cam155. These blood samples were drawn for clinical purposes at time points when the health status of the animal deteriorated and medical intervention was needed. Partially overlapping subgenomic fragments (1,100 bp to 3,950 bp in length) were amplified by PCR to obtain two full-length genome sequences of 9,899 bp for SIVcpz*Ptt*-04Cam155 and 9,870 bp for SIVcpz*Ptt*-09Cam155 infecting Cam155 in 2004 and 2009, respectively (Figure 1). Inspection of the deduced protein sequences of both SIVcpz*Ptt*-Cam155 sequences revealed open reading frames for *gag*, *pol*, *vif*, *vpr*, *tat*, *rev*, *vpu*, *env *and *nef *genes. To compare SIVcpz*Ptt*-Cam155 to previously characterized SIVcpz*Ptt *and SIVcpz*Pts *strains, we performed diversity plot analyses of concatenated sequences (data not shown) and phylogenetic tree analyses. The diversity plot and phylogenetic analyses (Figure 2) revealed that SIVcpz*Ptt-*Cam155 was a typical SIVcpz*Ptt *virus related across the entire genome to SIVcpz*Ptt-*Cam13 and SIVcpz*Ptt-*Gab1, infecting wild-caught chimpanzees from the south-western part of Cameroon and northern Gabon, respectively [2,3]. Mitochondrial DNA analysis on host DNA confirmed that Cam155 belongs to the *P. t. troglodytes *subspecies. As expected, the virus sequences obtained from Cam155 in 2004 and 2009 were very similar along the genome (Figure 2) with an average nucleotide similarity of 0.979. *pol *and *vif *were highly conserved after a five-year period, while LTR, *gag*, *nef*, and *env *regions were under higher selective pressure reflected by the accumulation of mutations. The coding region with the highest rate of mutations was in gp120, between V1 and V4 hypervariable loops. Analysis of the amino acid sequences in Pol revealed the absence of naturally present mutations typically associated with HIV drug resistance (according to HIVDB, ANRSV2009.07 and RegaV8.0.2 algorithms). It can also be noted that there were three and two copies of NF-kB enhancer in the 5'LTR of SIVcpz*Ptt*-04Cam155 and SIVcpz*Ptt*-09Cam155, respectively.

### Genetic variability of the hypervariable V1V4 env region over time

**Figure 3:**
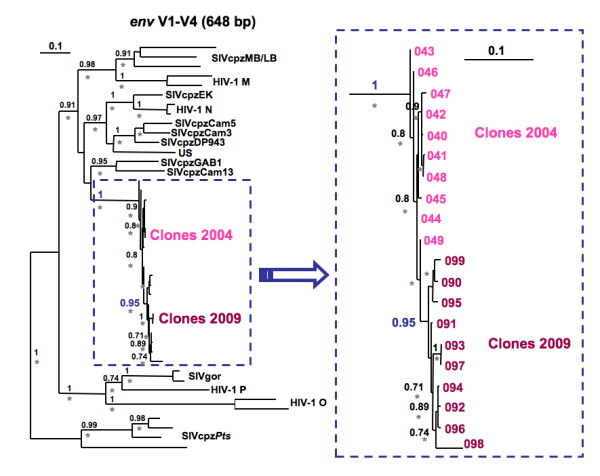
**Phylogenetic relationships of SIVcpz*Ptt*-Cam155 V1V4 *env *clones**. Analyses were performed using a codon nucleotide alignment of 648 bp, once the gaps discarded, of the ten clones of SIVcpz*Ptt*-Cam155 from 2004 and the ten ones from 2009 with previously published HIV-1/SIVcpz/SIVgor sequences. Phylogenetic analyses were run with both PhyML and Mr Bayes. Here presented in black and above the branches, the bootstraps > 0.70, and the grey stars below the branches are posterior probabilities > 0.80. Scale bars represent 0.1 replacements per site. On the right side is a zoom of the clones' phylogenetic relationships. The clone names 040 to 049 and 090 to 099 stand for SIVcpz*Ptt*-Cam155 clones from 2004 and 2009, respectively, indicated by 04 and 09 followed by the clone number.

To perform in-depth analysis on the variability of the envelope gene, we amplified a sub-genomic fragment spanning the V1V4 region (1,105 bp) for both 2004 and 2009 samples with specific primers. The amplified and gel-purified products were cloned and sequences of ten V1V4 molecular clones at each time point (2004 and 2009) were analyzed. The phylogenetic tree of V1V4 clones shows that strains from 2004 and 2009 form separate clusters according to their collection date (Figure 3), illustrating viral adaptation in its natural host over time. The calculations of dN and dS, with SNAP [24] of the different V1V4 *env *clones, showed a positive selective pressure (dN-dS >0) over the 1,000 bp with an important increase of non-synonymous substitutions between 2004 and 2009. Together, with a significant two-fold increased amino acid and nucleotide diversity (p < 0.00001) of the V1V4 region over a five-year period of infection (Table 2), these data confirm the important selective pressure exerted on SIVcpz*Ptt*-Cam155.

**Table 2:** Summary of SIVcpz*Ptt*-Cam155 amino acid and nucleotide diversities, sequence length and glycan shield of V1-V4 *env *clones in 2004 and 2009

	SIVcpz*Ptt*-04Cam155	SIVcpz*Ptt*-09Cam155	*p values (04 vs 09)*
**Diversity (aa) **(Min, Max) St Dev	**0.0471 **(0, 0.1035) 0.0364	**0.0884 **(0, 0.1569) 0.0512	** 8.8 10^**-06**^
**Diversity (nt) **(Min, Max) St Dev	**0.0237 **(0.0052, 0.0572) 0.0161	**0.0491 **(0, 0.0926) 0.0206	** 1.2 10^**-05**^
**Length (aa) **(Min, Max)	**332.6 **(330, 338)	**331.3 **(329, 335)	**0.21**
**# N-glyc sites **(Min, Max)	**17.1 **(16, 19)	**19.9 **(18, 21)	** 3.0 10^**-04**^

At each time point (2004 and 2009), the mean, minimum (Min), maximum (Max), and standard deviation (St Dev) of the pairwise amino acid (aa) and nucleotide (nt) diversities are shown. The mean, minimum, and maximum are also shown for V1-V4 amino acid length and the number of putative N-linked glycosylation sites. The p values were calculated by a Mann-Whitney test to assess the statistical differences between each mean in 2004 vs 2009 (** is highly significant).

**Figure 4:**
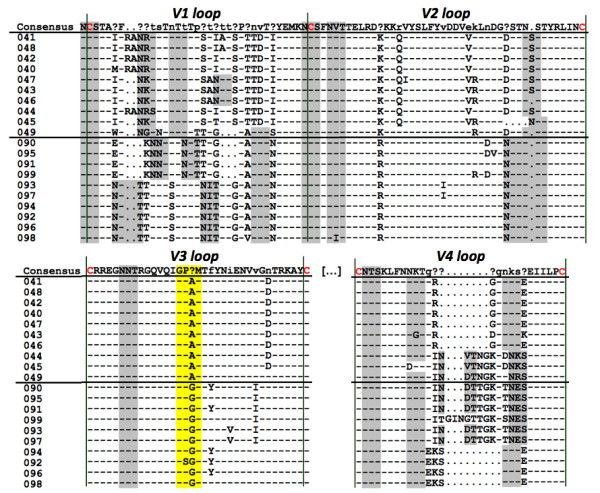
**Env hypervariable loop amino acid diversity of SIVcpz*Ptt*-Cam155 clonal sequences in 2004 and 2009**. The hypervariable loops V1, V2, V3 and V4 are analyzed. The alignment consensus of all clonal sequences is indicated at the top. The dots stand for gaps, dashes for the same amino acid as the consensus, the question marks in the consensus reveal no major amino acid in the alignment. On the left side, the clone names 040 to 049 and 090 to 099 stand for SIVcpz*Ptt*-Cam155 clones from 2004 and 2009, respectively. The glycosylation consensus motifs (NXT/S) are highlighted in grey, important cysteines in red and the V3 crown in yellow stressing the switch from 2004 to 2009.

Furthermore, the number of putative N-linked glycosylation sites (PNGS) in V1V4 *env *region increased significantly from an average of 17.1 to 19.9 between 2004 and 2009 (p < 0.0001) (Table 2). Notably, in SIVcpz*Ptt*-09Cam155 clones, an additional PNGS was observed in the V2 loop and in the majority of V1 sequences, and the glycosylation patterns of the V4 loop were variable according to indels (Figure 4). There was no significant difference in the SIVcpz*Ptt*-Cam155 *env *amino acid length between 2004 and 2009 (Figure 4). Particularly, the V2 loop remained stable in length over time, while an extension of the V2 loop has been associated with a slow disease progression [25,26], and a cycling pattern in V2 length was observed in the non-progressor SIVcpz*Pts*-Ant infected chimpanzee [7].

The different analyzed clones were all from the R5 phenotype, according to V3 net charges and the 11/25 rule [27]. In 2004, the V3 net charge of SIVcpz*Ptt*-04Cam155 was of 2 (<5). In 2009, an increase of the V3 net charge was observed (net charge = 3), but it was still inferior to the threshold (<5) associated with a switch from CCR5 to CXCR4 co-receptor use. An amino acid modification was observed in the V3 crown with a switch from GPAM (in 2004), mainly found in HIV-1 group N and its SIVcpz*Ptt *precursors, to a GPGM motif in 2009, found in the large majority of SIVcpz strains, with the exception of SIVcpz*Ptt *ancestors of HIV-1 group M which harbour a GPGQ/R crown (Figure 4). This amino acid change in a crucial position of the envelope and the conserved D to N mutation at position 29 on the V3 loop (Figure 4) are possibly due to an adaptation of the virus over time in response to host immune pressure.

## Discussion

To-date, data on the evolution of natural SIVcpz infection over time and on the impact of SIVcpz on chimpanzee populations are only available for the *P. t. schweinfurthii *subspecies from East-Central Africa and no data currently exist for representatives of the *P. t. troglodytes *subspecies, the natural reservoir of the ancestors of HIV-1 in humans. In this study, we describe for the first time the clinical observations and viral history over time in a naturally SIV infected *P. t. troglodytes *chimpanzee (Cam155/Ch-Go). The low CD4 counts observed in 2009, together with severe thrombocytopenia, weight loss and unusual frequent periods of infections with diverse pathogens, suggest a progressive SIV infection similar to HIV infection in humans, confirming previous observations that SIVcpz can be pathogenic in its natural host. Although, CD4 counts in 2004 and 2009 were measured with two different techniques, the observed decline in CD4 counts cannot be explained by potential different performances of the techniques on chimpanzee cells only. Moreover in 2009, CD4 counts of 1,256 CD4 cells/mm^3 ^were observed on another SIV negative chimpanzee from the same sanctuary versus 283 CD4 cells/mm^3 ^for Ch-Go with the same technique, and values reported in the literature for healthy SIV negative chimpanzees range also between 800 and 2,000 CD4 cells/mm^3 ^[23].

When applying the CDC and WHO classification systems to the clinical and biological data available for Cam155/Ch-Go and reported in this study, the evolution of the SIV infection in this *P. t. troglodytes *chimpanzee currently corresponds to pre-AIDS in humans, CDC B2 or WHO stage III. The viral loads fluctuated between 4 and 5 log_10 _copies/ml. Nevertheless, different techniques were used to measure viral loads in Cam155/Ch-Go at the different time points, and it cannot be excluded that the commercial HIV-1 viral load assays used in this study underestimated values for SIVcpz*Ptt*. The viral loads observed in Cam155/Ch-Go are in the range of the values observed for Ch-No (SIVcpz*Pts*-Ant), the other naturally SIVcpz infected chimpanzee, although from the *P. t. schweinfurthii *subspecies and with an apparent non-progressive SIV infection [8,28]. In the absence of a specific SIVcpz viral load test, comparisons over time or with other experimentally or naturally infected animals are difficult. Moreover, in Ch-No, viral loads fluctuated over time from 3.4 to 5.8 log_10 _copies/ml and could differ by more than 1 log according to the technique used [8]. In addition to PCR, quantitative viral isolations have also been done from different plasma and PBMC dilutions for Ch-No, and important fluctuations have been observed over time, although there was no correlation at all between titres of infectious virus in plasma and viral load measured by PCR [8]. No other data on viral load observed in natural SIVcpz infections are readily available for comparison but, for natural non-pathogenic SIVsm and SIVagm infections in mangabeys and African green monkeys, viral loads are also generally high [29,30]. In contrast, in chimpanzees that were previously experimentally infected with HIV-1, plasma viral loads were undetectable or very low, except for the few animals that progressed to AIDS 4 to 18 years post-inoculation and for whom viral loads increased over time and could reach up to 6 log_10 _copies/ml [16]. CD4 decline, severe thrombocytopenia, increased plasma viral loads and occurrence of opportunistic infections were also observed in the HIV-1 experimentally infected chimpanzees that developed AIDS in the Yerkes Primate Center [31]. The animals that progressed faster to AIDS underwent superinfections with 2 or 3 strains, which was the case for the first animal (C499) that was reported with AIDS, or were infected with the pathogenic strain of this latter animal.

The naturally SIV infected *P. t. schweinfurthii *chimpanzee, Ch-No is still alive and in good health today, more than 20 years later, despite the relatively high plasma viral load and a severe and permanent thrombocytopenia that occurred approximately at age 7 [28]. Thrombocytopenia was also seen in the experimentally infected chimpanzees with AIDS and is observed in humans and macaques with AIDS [12,32]. Whether the asymptomatic period for natural SIVcpz is longer than for HIV in humans, or whether differences in incubation periods exist like in humans (i.e. rapid versus long-term progressors), is not known. Thus, it cannot be excluded that the SIVcpz*Pts*-Ant infected chimpanzee may still develop a progressive infection.

Given the young age of Cam155 at seizure (1.5 years old), the chimpanzee was likely infected through vertical mother-to-child SIV transmission, as chimpanzees are not sexually active before the age of 8; however, horizontal transmission by blood contact (e.g. biting injuries) cannot be entirely excluded. In humans, *in utero *infected newborns develop AIDS more rapidly compared to those infected after birth [33], but survival rates and disease progressions in vertically HIV-1 infected infants can be variable [34]. Recent studies on SIV pathogenicity in wild East African chimpanzees show a higher mortality rate among infants born to SIV positive mothers [13,35]. The majority of the other known SIVcpz positive captive chimpanzees were most likely also infected through mother-to-child transmission, because they were all less than 3-4 years old at time of rescue (Additional file 1: Table S1) [1-3,6,36,37]. Although they had no signs of AIDS at the time of diagnosis, some had chronic lymphadenopathy like Gab1 and cpz-US, or thrombocytopenia as Ch-No. Some died suddenly from acute infections (Cam5 and Cam13), as shown in Additional file 1: Table S1 summarizes the history of the previously reported SIVcpz positive captive animals; however, whether this was related to the SIV infection and an eventual degradation of the immune system is not known.

Phylogenetic analyses revealed that SIVcpz*Ptt*-Cam155 fell within the radiation of the SIVcpz*Ptt *group of viruses, as part of a clade including all other SIVcpz*Ptt *strains, as well as HIV-1 groups M and N. However, SIVcpz*Ptt-*Cam155 clustered most closely with SIVcpz*Ptt-*Gab1 from northern Gabon and SIVcpz*Ptt-*Cam13 from southwest Cameroon. We previously reported phylogeographic clustering of SIVcpz*Ptt *strains in Cameroon, and observed high genetic diversity within small geographic areas. Although the geographic origin of this animal is not precisely known, it most likely originated around the Dja Reserve in south central Cameroon. The SIVcpz*Ptt*-Cam155 sequence further illustrates the high genetic diversity among SIVcpz*Ptt *strains in this area [5].

Our data demonstrate an important diversification and mutation rate of SIVcpz*Ptt*-Cam155 over time, with nucleotide and amino acid diversity doubling in 5 years in the envelope, and an evolution of the putative envelope structure leading to escape mutants. Particularly, V1 and V4 loops were highly variable, as similarly observed in experimentally SIV infected macaques during progression to simian AIDS [38]. Moreover, variability in V4 region is associated with modification of CD4 binding and plays a key role in the swarming nature of gp120 [39]. V3 was modified in its crown and V2 was stable, in contrast to SIVcpz*Pts-*Ant [7] or slow disease progressors [25,26]. The progressive diversification of HIV in untreated infected humans underlies its ability to evade immunologic selective pressure, but this diversification may also be responsible for disease progression and destruction of immune system [40]. Overall the evolutionary rate of HIV-1 slows down over time and seems to be correlated with the slope of the CD4 cell decline. Considering two time points, the SIVcpz*Ptt*-Cam155 V1V4 nucleotide diversities five years apart (0.0237 in 2004 and 0.0491 in 2009) fit the trends of viral diversification across HIV-1 infected humans in diverse studies [41,42], and the theoretical curve established by Lee *et al. *describing the evolution of C2V5 HIV diversity over time [43]. In chimpanzees experimentally infected with HIV-1 [17], a higher viral diversity was seen in the progressor chimpanzees vs. the non-progressors; however, it has to be noted that these data originated from animals inoculated with two distinct HIV-1 strains, and recombination between the different strains could have biased the overall diversity observed over time. We also observed an increase of putative N-linked glycosylation sites over time in SIVcpz*Ptt*-Cam155 envelope. Specific genetic modifications leading to the acquisition of PNGS were shown to result in an evolving protective glycan shield [44] and to be a characteristic of escape mutants since it reduces protein epitope exposure and thus facilitates viral evasion of antibody neutralization [45].

## Conclusion

Our study provides additional evidence that SIVcpz infection is associated with clinical disease in chimpanzees and that it affects both Eastern and Central African chimpanzee subspecies. We also showed SIVcpz*Ptt *viral diversification and adaptation in its natural host. Only a future detailed and regular clinical, immunological and virological follow-up on naturally infected animals over time will allow us to determine to what extent SIVcpz infection resembles that of HIV-1 in humans. Given the poor health status of the animal described in this study, the administration of antiretroviral therapy may be necessary in the near future in order to avoid further progression to AIDS and to ensure the lengthened survival of this chimpanzee. Although studying the impact of SIV infection in wild chimpanzees is difficult because they live in isolated forest regions, efforts should be made to monitor health status in ape populations to find out whether SIV infection plays a role in population decline, in addition to habitat destruction, poaching and other disease pressures, such as Ebola virus. No data are currently available on the pathogenicity of SIV in gorillas. Nevertheless, as gorillas are infected with SIVgor, most likely through cross-species transmission of SIVcpz from chimpanzees, it is probable that SIVgor also has a negative impact on the health of gorillas in the wild. The fact that chimpanzees naturally infected with SIV have been rescued (7 individuals between 1988 and 2008) further indicates that humans hunting apes are exposed to SIVs and are at risk for cross-species transmission of SIVcpz. Such cross-species transmissions present a risk of potential emergence of new strains in the human population, which could make HIV treatment and vaccine development more difficult.

## Methods

### Serological testing of the SIVcpz*Ptt*-Cam155 infected chimpanzee

In November 2003, a 1.5 year old male chimpanzee (Cam155) was seized by the Ministry of Environment and Forestry from the area around the Dja reserve, south central Cameroon. During his quarantine in December 2003, the animal was screened for SIV infection with HIV screening tests, i.e. a rapid test (Multispot HIV-1/HIV-2 Rapid Test (Bio-Rad, Marnes-la-Coquette, France)), an indirect ELISA (HIV-1 and HIV-2 GenElavia Mixt (BioRad)), and a competitive Elisa (Wellcozyme rec HIV-1 (Murex/Abbott, Dartford, Kent, UK)) [46]. These reactions were confirmed by western-blot analysis (New Lav Blot HIV-1/2, Bio-Rad). Serological tests for other viral infections were also performed: hepatitis A virus (HAV) by Monolisa™ anti HAV IgM EIA (BioRad), HBV using Monolisa™ Ag HBS plus (BioRad), and HCV using Monolisa™ anti-HCV plus, version 2 (BioRad), and simian T-lymphotropic virus using Platellia™, HTLV-1/2 (BioRad).

### Full-length sequence of the SIVcpz*Ptt*-Cam155 strain and envelope clones

Total DNA was extracted from leftover buffy coat or PBMCs using the QIAamp blood kit (Qiagen, Courtaboeuf, France). Full-length sequences of the SIVcpz strains infecting Cam155 in 2004 (9,899 bp) and 2009 (9,870 bp) were generated by amplifying partially overlapping subgenomic fragments (1,100 bp to 3,950 bp in length) using SIVcpz/HIV-1 consensus primers and SIVcpz*Ptt*-Cam155 specific primers (Additional file 2: Table S2, Figure 1). All PCR reactions were performed with the Expand Long Template PCR system (Roche Diagnostics, Indianapolis, IN) and PCR conditions were as previously described [5]. The resulting amplification products were gel purified (Geneclean Turbo Kit, Qbiogene, Carlsbad, CA) and directly sequenced on an automated sequencer (3130xl Genetic Analyser, Applied Biosystems, Foster City, CA), except for the V1V4 *env *region for which clonal sequences were necessary since chromatograms from direct sequence analysis could not be resolved. We amplified a fragment spanning the V1V4 *env *region (1,105 bp) of SIVcpz*Ptt*-Cam155 from 2004 and 2009 with specific primers (Additional file 2: Table S2, Figure 1). The amplified and gel purified products were cloned following the manufacturer's instructions (pGEM-T easy vector system II, Promega, Madison, WI) and ten SIV clones for each time point were sequenced to analyse the viral envelope diversity. SIVclone041 and SIVclone091 were arbitrarily selected to cover the V1V4 *env *region in the full-length sequences of SIVcpz*Ptt*-04Cam155 and SIVcpz*Ptt*-09Cam155 respectively.

### Phylogenetic and genetic diversity analyses

Phylogenetic analyses were performed for each main gene of the SIV genome, but the Pol region was divided in two fragments according to the recombination point observed for other SIVcpz and HIV-1 N viruses [4]. SIVcpz*Ptt*-04Cam155 and SIVcpz*Ptt*-09Cam155 Gag, Pol1, Pol2, and Env amino acid (aa) sequences were compared to previously published SIVgor, SIVcpz and HIV-1 references. Sequences were aligned using Mega4 [47] and where necessary, minor manual adjustments were performed. Sites that could not be unambiguously aligned or contained a gap in any sequence were excluded from the analyses. In the end, 422 amino acids were examined for Gag analysis, 700 aa for Pol1, 251 aa for Pol2, and 673 aa for Env. Maximum likelihood (ML) trees were constructed using PhyML http://www.atgc-montpellier.fr/ with 1,000 bootstrap replicates [48]. Phylogenies were also inferred by the Bayesian method [49], implemented in MrBayes version 3.1 [50], run for 3,000,000 generations, and trees sampled every 100 generations, the first 25% being discarded as burn-in. Parameters were examined with the Tracer program http://tree.bio.ed.ac.uk/software/tracer/. For the Gag, Pol2, and Env regions, the Jones, Taylor and Thornton (JTT) model for protein evolution [51] with a gamma distribution [52] across sites was the most appropriate model according to TOPALI [53] and Bayesian estimation [50]. Nevertheless, for Pol1 analysis, the RtREV model [54] was found to be the best model. Phylogenetic analyses were also performed for the V1V4 region using a codon nucleotide alignment of 648 bp, once the gaps discarded. The best evolution model was the general time-reversible (GTR) model with a gamma distribution across sites. The phylogenetic analyses were run with both PhyML and Mr Bayes with the same characteristics as shown above.

Diversity plots were made using a sliding window of 300 nucleotides and moved in steps of 50 residues. The cumulative number of non-synonymous and synonymous nucleotide substitutions (dN and dS) was estimated using SNAP [24]. Viral diversity of SIVcpz*Ptt*-Cam155 V1V4 *env *region in 2004 and 2009 was determined by calculating pairwise nucleotide and amino acid distances between V1V4 *env *clones with Mega4 [47] with the Tamura and Nei method [55] and the Gamma distance method, respectively. At each time point (2004 and 2009), the mean, minimum, maximum, and standard deviation of amino acid and nucleotide diversities were calculated. Amino acid sequence length and putative N-linked glycosylation diversity, which were five years apart, were compared. P values were estimated by a Mann-Whitney test to assess the statistical differences between 2004 vs 2009 viral diversity, length variation and PNGS.

#### Accession numbers

GenBank accession numbers for complete genome sequences used in comparative analyses are as follows: SIVcpz*Pts*: ANT (U42720), TAN1 (AF447763), TAN2 (DQ374657), TAN3 (DQ374658); SIVcpz*Ptt*: MB897 (EF535994), LB7 (DQ373064), MB66 (DQ373063), EK505 (DQ373065), CAM5 (AJ271369), DP943 (EF535993), CAM3 (AF115393), US (AF103818), GAB1 (X52154), CAM13 (AY169968); SIVgor: CP684 (FJ424871), CP2135 (FJ424863); HIV-1 group M: subtype A U455 (M62320), subtype B HXB2 (K03455); HIV-1 group N: YBF106 (AJ271370), YBF30 (AJ006022); HIV-1 group O: MVP5180 (L20571), ANT70 (L20587); HIV-1 group P: RBF168 (GQ328744).

The two complete genomes SIVcpz*Ptt*-04Cam155 and SIVcpz*Ptt*-09Cam155 are available under accession numbers [EMBL:FR686510-1], and the 20 envelope clones spanning the V1V4 *env *region are under accession numbers [EMBL:FR686512-31].

## Competing interests

The authors declare that they have no competing interests.

## Authors' contributions

LE performed all molecular, phylogenetic and diversity analyses with the contribution of AA. EN, NW, ML, and UT initiated and coordinated collaboration between Yaoundé Zoo/Sanctuary and the laboratory of virology of Centre Pasteur du Cameroun (CPC). EN supervised all activities related to initial diagnosis, virological and immunological monitoring with YF, AA and DR. AFA, ML, AN, CD and EM continued clinical and laboratory monitoring. GTB rescued the animal and is involved in daily care of Cam155/Ch-Go. ED and EM provide advice on clinical monitoring. LE, AA, ML and MP wrote the manuscript. AA, ML, NW and MP coordinated the study.

## Supplementary Material

Additional file 1 **Table S1 Naturally SIVcpz infected captive chimpanzees reported in the literature**. Table summarizing the history of the 9 SIVcpz positive captive chimpanzees, with details on their capture, their SIV strain, their current status and remarks of interest. Click here for file

Additional file 2 **Table S2 Primer sets used to amplify SIVcpz*Ptt*-04Cam155 and SIVcpz*Ptt*-09Cam155 PCR fragments**. Click here for file
