# Supplementary material for: Characterization of a new simian immunodeficiency virus strain in a naturally infected Pan troglodytes troglodytes chimpanzee with AIDS related symptoms
Source: Retrovirology. 2011 Jan 13;8:4. doi: 10.1186/1742-4690-8-4 (PMC3034674; doi:10.1186/1742-4690-8-4)
Supplement: Additional file 1 — Table S1 Naturally SIVcpz infected captive chimpanzees reported in the literature. Table summarizing the history of the 9 SIVcpz positive captive chimpanzees, with details on their capture, their SIV strain, their current status and remarks of interest. [file 1742-4690-8-4-S1.PDF]

Additional Table 1. Naturally SIVcpz infected captive chimpanzees reported in the literature.

Id[a]	Sub-species[b]	Year of rescue	Age at rescue[c]	Sex[d]	Country of rescue[e]	Locality	SIVcpz strain (seq[f])	Current status	Remarks	Ref.
Cam155	*P.t.t.*	2003	1.5	M	Cameroon	Dja region	SIVcpzPtt -Cam155 (FL)	Alive at 8.4 yrs	Profound thrombocypenia, recurrent *Blantidium coli* infections, recurrent respiratory illness, oral candidosis, immune depression	This study
Cam13	*P.t.t.*	2001	2	M	Cameroon	Littoral province	SIVcpz*Ptt* -Cam13 (FL)	Deceased in 2002 from massive parasite infection		[3]
Cam5	*P.t.t.*	1998	1	F	Cameroon	Central province	SIVcpz*Ptt* -Cam5 (FL)	Deceased in 1998 upon arrival from severe diarrhea	No clinical signs associated with AIDS	[1]
Cam4	*P.t.e.*	1993	2.5	M	Cameroon	Southwest province	SIVcpz*Pte* -Cam4 (P)	Alive at 20 yrs	Presumed recipient of cage transmission since housed with Cam3. Healthy apart from episodes of fever and diarrhea	[1]
Cam3	*P.t.t.*	1992	1	M	Cameroon	Dja Forest reserve	SIVcpz*Ptt* -Cam3 (FL)	Deceased in 1998 suddenly from subaccute pneumonia	Housed 5 years with Cam4 in the same enclosure before testing	[1, 36]
Gab1	*P.t.t.*	1988	0.5	F	Gabon	Oyem region	SIVcpz*Ptt* -Gab1 (FL)	Deceased in 1990 from unknown causes	Kept as a pet, no haematological abnormalities but history of chronic lymphadenopathy	[56]
Gab2	*P.t.t.*	1988	2	F	Gabon	Macatama-goye village	SIVcpz*Ptt* -Gab2 (P)	Deceased in 1988 upon arrival from shot wounds inflicted by hunters	No adenopathy or haematological disorders	[2]
Noah	*P.t.s.*	1987	2	M	DRC	not known	SIVcpz*Pts* -Ant (FL)	In good health today at 25 yrs	No signs of immunodepression but a profond and permanent thrombocytopenia. Followed-up clinically and virologically for 7 years (age of 4 to 11 years)	[6, 7]
Marilyn	*P.t.t.*	In US in 1963	4 in the US	F	not known	not known	SIVcpz*Ptt* -US (FL)	Deceased in 1985 giving birth from pneumonia and toxic sequelae to the stillbirths	Necropsy revealed generalised lymphadenopathy, erythrophagocytosis, extramedullary haematopoieses, etc.	[37]

a, Id, Identification
b, *P.t.t., Pan troglodytes troglodytes; P.t.s., Pan troglodytes schweinfurthii; P.t.e., Pan troglodytes ellioti.* Subspecies determined by mitochondrial DNA analyses.
c, Estimated age (in years) at rescue.
d, F, female; M, male.
e, DRC, Democratic Republic of Congo.
f, SIVcpz sequence available; FL, full-length genome; P, partial genome.
