# Supplementary material for: Characterization of a new simian immunodeficiency virus strain in a naturally infected Pan troglodytes troglodytes chimpanzee with AIDS related symptoms
Source: Retrovirology. 2011 Jan 13;8:4. doi: 10.1186/1742-4690-8-4 (PMC3034674; doi:10.1186/1742-4690-8-4)
Supplement: Additional file 2 — Table S2 Primer sets used to amplify SIVcpzPtt-04Cam155 and SIVcpzPtt-09Cam155 PCR fragments. [file 1742-4690-8-4-S2.PDF]

Additional Table 2. Primer sets used to amplify SIVcpz*Ptt*-04Cam155 and SIVcpz*Ptt*-09Cam155 PCR fragments.

a. For SIVcpz*Ptt*-04Cam155 complete genome

Fragment name [a]	Gene	Primers [b]		Amplicon (bp) [c]
A	*pol-gp41*	CPZ-A-F1	CCAGCNCACAAAGGNATAGGAGG	4300
		CPZ-A-R1	AACGACAAAGGTGAGTATCCCTGCCTAA	
		CPZ-A-F2	GGAAGTGGATACTTAGAAGCAGAAGT	3824
		CPZ-A-R2	TCCTACTATCATTATGAATATTTTTATATA	
B	*gag-pol*	CPZ-B-F1	ATGGGWGCGRGDGCGTC	4211
		CPZ-B-R1	ACBACYGCNCCTTCHCCTTTC	
		CPZ-B-F2	TRAAACAYHTRGTWTGGGCAAG	3947
		CPZ-B-R2	CCAATYCCYCCYYTTYKYTTAAAATT	
C	*env-gag*	CPZ-C-F1	TCTTAGGAGCAGCAGGAAGCACTATGGG	4333
		CPZ-C-R1	CCAAAGAGKGATTTSAGGGA	
		CPZ-C-F2	ACAATTATTGTCTGGTATAGTGCAACAGCA	3684
		CPZ-C-R2	TCYTKTCCACAYYTCCARCA	
D	*env* (V1-V4)	Cam155-D-F1	AATATCTGGGCCACGCAAGCAT	1200
		Cam155-D-R1	AGTTCGCCTCTTTGCCTTCTGT	
		Cam155-D-F2	TGTGGGATAATCCTATGGTGTC	1105
		Cam155-D-R2	TTTCCCTCTTCACGTGCCAACA	

b. For SIVcpz*Ptt*-09Cam155 complete genome

Fragment name [a]	Gene	Primers [b]		Amplicon (bp) [c]
a	*gag-pol*	CPZ-a-F1	ATGGGWGCGRGDGCGTC	4211
		CPZ-a-R1	ACBACYGCNCCTTCHCCTTTC	
		CPZ-a-F2	TRAAACAYHTRGTWTGGGCAAG	3947
		CPZ-a-R2	CCAATYCCYCCYYTTYKYTTAAAATT	
b	*env-nef*	CPZ-b-F1	TCTTAGGAGCAGCAGGAAGCACTATGGG	1338
		CPZ-b-R1	CCCHTCCAGTCCYCCCTTTTC	
		CPZ-b-F2	ACAATTATTGTCTGGTATAGTGCAACAGCA	1200
		CPZ-b-R2	CAGTCCYCCCTTTTCTTTYAAAAA	
c	*pol- vpu*	CPZ-c-F1	TGGTGGDCWGANTAYTGGCA	2102
		CPZ-c-R1	CCARGGSKSYANNTYAGGATCTAYTGGHTCCAT	
		CPZ-c-F2	CATGTRGCHAGTGGNTWCMTAGARGCAGARGT	1396
		CPZ-c-R2	ATBCCTATTCTRCTATGTTGRCABCCAA	
d	*nef-gag*	CPZ-d-F1	TAGTAAMAAAAGTTAGGCAGGG	3260
		CPZ-d-R1	GCTTCWGCTARNACYCTWGCCTTATG	
		CPZ-d-F2	ADGAAGAAGTAGGYTTYCCAGT	2100
		CPZ-d-R2	TCCCAHTCNGCDGCTTCYTCATTGAT	
e	*env*	CPZ-e-F1	ACAGTATWYTATGGRGTHCCAGTNTGG	2797
		CPZ-e-R1	CAGTCCYCCCTTTTCTTTYAAAAA	
		CPZ-e-F2	TGTGGGATAATCCTATGGTGTC	1799
		CPZ-e-R2	TCCTACTATCATTATGAATATTTTTATATA	
f	*vif-env*	Cam155-f-F1	CTGCAGGAGAAAGAATCATAGA	3300
		CPZ-f-R1	TCCTACTATCATTATGAATATTTTTATATA	
		Cam155-f-F2	GCCACCCAGAGAACCATACAAT	2014
		Cam155-f-R2	TTTCCCTCTTCACGTGCCAACA	
g	*env* (V1-V4)	Cam155-g-F1	AATATCTGGGCCACGCAAGCAT	1200
		Cam155-g-R1	AGTTCGCCTCTTTGCCTTCTGT	
		Cam155-g-F2	TGTGGGATAATCCTATGGTGTC	1105
		Cam155-g-R2	TTTCCCTCTTCACGTGCCAACA	

[a] Fragment names are as defined in Figure 1.

[b] CPZ primers were designed according to SIVcpz/HIV-1 consensus sequences [5, 57]; Cam155 primers were specifically designed according to SIVcpz*Ptt*-Cam155 sequence. F1, first-round forward primer; F2, second-round forward primer; R1, first-round reverse primer; R2, second-round reverse primer. K=G/T, M=A/C, R=A/G, W=A/T, Y=C/T, B=C/G/T, D=A/G/T, H=A/C/T and N=A/C/T/G.

[c] Size of PCR amplicons.
